# Myocardial Involvement in COVID-19: an Interaction Between Comorbidities and Heart Failure with Preserved Ejection Fraction. A Further Indication of the Role of Inflammation

**DOI:** 10.1007/s11897-021-00509-y

**Published:** 2021-04-22

**Authors:** Gregorio Zaccone, Daniela Tomasoni, Leonardo Italia, Carlo Mario Lombardi, Marco Metra

**Affiliations:** 1 Cardiology, ASST Spedali Civili di Brescia, Brescia, Italy; 2 Department of Medical and Surgical Specialties, Radiological Sciences, and Public Health, University of Brescia, Brescia, Italy

**Keywords:** COVID-19, SARS-CoV-2 infection, Heart failure, Heart failure with preserved ejection fraction, HFpEF

## Abstract

**Purpose of the review:**

Coronavirus Disease 2019 (COVID-19) and cardiovascular (CV) disease have a close relationship that emerged from the earliest reports. The aim of this review is to show the possible associations between COVID-19 and heart failure (HF) with preserved ejection fraction (HFpEF).

**Recent findings:**

In hospitalized patients with COVID-19, the prevalence of HFpEF is high, ranging from 4 to 16%, probably due to the shared cardio-metabolic risk profile. Indeed, comorbidities including hypertension, diabetes, obesity and chronic kidney disease — known predictors of a severe course of COVID-19 — are major causes of HFpEF, too. COVID-19 may represent a precipitating factor leading to acute decompensation of HF in patients with known HFpEF and in those with subclinical diastolic dysfunction, which becomes overt. COVID-19 may also directly or indirectly affect the heart. In otherwise healthy patients, echocardiographic studies showed that the majority of COVID-19 patients present diastolic (rather than systolic) impairment, pulmonary hypertension and right ventricular dysfunction. Such abnormalities are observed both in the acute or subacute phase of COVID-19. Cardiac magnetic resonance reveals myocardial inflammation and fibrosis in up to the 78% of patients in the chronic phase of the disease.

**Summary:**

These findings suggest that COVID-19 might be a novel independent risk factor for the development of HFpEF, through the activation of a systemic pro-inflammatory state. Follow-up studies are urgently needed to better understand long-term sequelae of COVID-19 inflammatory cardiomyopathy.

## Introduction

Since the first identification of Severe Acute Respiratory Syndrome Coronavirus 2 (SARS-CoV-2), the Coronavirus Disease 2019 (COVID-19) pandemic quickly spread around the world, becoming a major challenge for health systems [1]. From the earliest reports, a close relationship emerged between COVID-19 and cardiovascular (CV) disease [2••]. On one hand, COVID-19 patients with pre-existing CV comorbidities have a worse outcome, on the other hand, COVID-19 may affect the heart causing myocardial injury and CV complications. Recent observations suggested that COVID-19 may be associated with both systolic and diastolic dysfunction and heart failure (HF) [3, 4]. The hypothesis that COVID-19 may cause heart failure with preserved ejection fraction (HFpEF), through direct viral injury or the indirect damage caused by immune reaction, has been raised. Moreover, COVID-19 may exacerbate HF symptoms in patients with known HFpEF or reveal subclinical HFpEF [5].

Hereby, we summarize the available literature regarding the evidences of myocardial damage both during the acute phase of the infection and as a long-term consequence of COVID-19, possibly leading to HFpEF. We will also discuss the intersection between COVID-19 and HFpEF, focusing on the shared cardiometabolic risk profile and the common inflammatory pathophysiology.

## Before COVID-19 and HFpEF: Risk Factors for Both

Many comorbidities, which are common in the most severe cases of COVID-19, are major causes or comorbidities of HFpEF, too, so that they may likely contribute to new onset HF in these patients. The clinical presentation of COVID-19 is extremely variable, ranging from an asymptomatic or pauci-symptomatic disease to severe pneumonia which can eventually lead to severe acute respiratory distress syndrome (ARDS) and death. Since the first Chinese reports, it appeared clear that older age and pre-existing comorbidities, namely hypertension, diabetes, and CV disease are highly prevalent in hospitalized patients, and are associated with a more severe course of the disease and higher mortality rates [6–8]. Afterwards, similar data were reported in other countries [9]. In a multicentre study conducted in Northern Italy, including 1591 patients requiring intensive care unit (ICU) admission, hypertension, CV disease and diabetes affected the 49, 21 and 17% of the cases, respectively [10]. Hypertension represented the most frequent comorbidity in COVID-19 patients, probably due to its high prevalence in the general population. Diabetes was more frequent in those with a severe course of the disease, rather than in patients with a mild form of COVID-19 (Odds Ratio [OR] 2.49, 95% confidence interval [CI] 1.70 to 3.64) [11]. Both hypertensive and diabetic patients had a greater risk of adverse outcome, compared to the overall population [12–15].

Studies from the USA highlighted also the high proportion of obesity as a comorbid condition in COVID-19. This was previously less reported probably due to its different prevalence in Asian countries [16, 17]. An increase in body mass index (BMI) was a strong predictor of hospitalization (for BMI >40 kg/m2: OR 2.5, 95% CI 1.8–3.4) [18], and was associated with higher rates of mechanical ventilation (for BMI >35 kg/m2: OR 7.36, 95% CI 1.63–33.14) [19]. Of note, most hospitalized patients with COVID-19 were old, and this could have influenced the burden of comorbidities. Nonetheless, in a population of 3222 young adults (aged 18–34 years), requiring hospitalization, morbid obesity, hypertension, and diabetes were common and conferred greater risks of adverse events [20]. Simonnet et al. showed that obesity remains an independent risk factor for adverse outcome in patients with COVID-19, even after adjustment for age, sex, and other variables [19]. Chronic kidney disease (CKD) represents another risk factor for the development of a severe form of COVID-19 and in-hospital death [21, 22].

## Before COVID 19: Pre-existing HF as a Risk Factor for Severe Disease

The prevalence of HF history in patients hospitalized for COVID-19 ranged from 3.3% to 10.1% in earlier reports [18, 23, 24]. A higher proportion (13%) has been recently reported in an Italian multicentre study, including 692 patients from 13 cardiology centres, which were temporally turned into COVID-19 units. In this cohort, 26% of HF patients had HFpEF [25], a percentage that seems lower compared to data reported in pre-COVID-19 times, with HFpEF accounting for more than half of all HF patients [26]. However, in a larger retrospective analysis including 6439 patients admitted for COVID-19 in New York City, HFpEF was reported in 4% of the overall population, corresponding to 59% of HF population [27]. Prevalence, comorbidities and outcomes of HFpEF in patients with COVID-19 are summarized in Table 1 [25, 27–29].

**Table 1:** Prevalence, comorbidities and outcomes of COVID-19 patients with history of HFpEF

Study	Design	Patients, *n*	HFpEF, %	Male, %	Age, years	Comorbidities (%)	Outcomes
Tomasoni et al. [25]	Retrospective	692	4	73.1	72.3 ± 12.3	HTN (76.9), DM (42.3), Obesity (-) CKD (61.6)	Higher mortality rate; increased risk of in-hospital complications
Alvarez-Garcia et al. [27]	Retrospective	6439	4	47.6	74.1 ± 12.5	HTN (91.6), DM (66.8), Obesity (-) (42.0), CKD (44.0)	2-fold higher mortality; increased risk of in-hospital complications
Raad et al. [28]	Retrospective	437	16.7	62.5	80.5 ± 11.6	HTN (94.4), DM (58.3), CKD (61.1)	2.6-fold higher mortality; increased risk of in-hospital complications
Li et al. [29]	Retrospective	157	15.3	-	-	HTN (-), DM (-), Obesity (-), CKD (-)	Higher mortality rate

*CKD*, chronic kidney disease; *DM*, diabetes mellitus; *HFpEF*, Heart Failure with Preserved Ejection Fraction; *HTN*, hypertension

Patients with previous HF had higher mortality rates [23, 25, 27]. The association between HF and mortality remained significant after adjustment for variables associated with COVID-19 and HF severity in several multivariable models. Importantly, the association was not modified by left ventricular ejection fraction (LVEF) [25, 27]. Raad et al. found that HFpEF was an independent predictor of mortality in patients admitted for COVID-19 with an adjusted OR of 2.6 (95% CI, 1.4–4.8) [28].

Patients with a history of HF were also more susceptible to develop acute HF [23, 25]. About one-third of patients with HF history had an acute decompensation of HF, regardless of LVEF [25]. Acute viral infections are known precipitant factors for acute exacerbation of HF [30]. On the other hand, almost a half of acute HF events of these series were not preceded by a HF history [23, 25], suggesting that further mechanisms may be involved in the cause-effect relationship between COVID-19 and HFpEF.

## During COVID-19: Myocardial Involvement in the Acute Phase

Evidence of myocardial damage during COVID-19 has been reported in various forms, including elevated cardiac biomarkers (troponin and natriuretic peptides), imaging abnormalities (echocardiography or cardiac magnetic resonance), and pathological histological findings. Current data are summarized in Table 2 [31–43].

**Table 2:** Evidence of myocardial damage during COVID-19 (acute phase) and after recovery (subacute and chronic phase)

Study	Design	Patients, *n*	Acute/subacute/chronic phase	Modality	Findings
Szekely et al. [31]	Prospective	100	Acute	Echocardiography	RV dilatation/dysfunction LV diastolic dysfunction
Mahmoud-Elsayed et al. [32]	Retrospective	74	Acute	Echocardiography	RV dilatation/dysfunction
Goerlich et al. [33]	Retrospective	73	Acute	Echocardiography	Elevated LV filling pressure, PH
Baycan et al. [34]	Prospective	100	Acute	Echocardiography	LV-GLS and RV-LS decrease
Lassen et al. [35]	Prospective	214	Acute	Echocardiograohy	LV-GLS, RV strain and TAPSE reduction
Ojha et al. [36•]	Systematic review	199	Acute	CMR	T1 and T2 abnormalities, edema on T2/STIR and LGE
Escher et al. [37]	Retrospective	104	Acute	EMB	Myocardial inflammation
Tavazzi G et al. [38]	Case report	1	Acute	EMB	Myocardial inflammation
Pietsch et al. [39]	Case report	1	Acute	EMB	Myocardial inflammation
Puntmann et al. [40]	Prospective	100	Subacute	CMR	Raised T1 and T2, LGE
Brito et al. [41]	Cross-sectional	54	Chronic	Echocardiograohy	GLS reduction
Rajpal et al. [42]	Prospective	26	Chronic	CMR	Myocarditis, LGE
Huang et al. [43]	Retrospective	26	Chronic	CMR	Raised T1, T2 and ECV

*CMR*, cardiac magnetic resonance; *ECV*, extracellular volume; *EMB*, endomyocardial biopsy; *GLS*, global longitudinal strain; LGE, late gadolinium enhancement; *LS*, longitudinal strain; *LV*, left ventricular; *PH*, pulmonary hypertension; *RV*, right ventricular; *STIR*, short tau inversion recovery; *TAPSE*, tricuspid annular plane systolic excursion

Troponin elevation was described in a variable proportion of patients hospitalized for COVID-19 with a range from 20% to 45% [44–46]. Elevated troponin levels have negative prognostic implications and their role was independent from other variables, including a history of HF, in some series [44–46].

Few cases of COVID-19-related acute myocarditis, presenting with severe reduction in LVEF were described [47, 48]. However, recent data showed that left ventricular (LV) systolic function is not compromised in the majority of patients affected by COVID-19, and the most frequent finding was an impairment of right ventricular (RV) function and LV diastolic function [31]. Among 100 consecutive patients diagnosed with COVID-19, 32% had normal echocardiography at presentation; RV dilatation and dysfunction were observed in 39% of patients, LV diastolic dysfunction in 16%, while reduced LVEF was reported only in less than 10%. Impairment of RV function was related with elevated troponin levels and clinical deterioration [31]. Similar results came from other smaller series [32, 49]. Goerlich et al. found LV diastolic impairment with elevated LV filling pressures (E/e’ ratio) in a quarter of patients admitted for COVID-19. They also reported an association with higher estimated RV systolic pressure (E/e’ 12.6 [8.7–15.7] versus 8.2 [6.6–9.9], *p* < 0.001 in patients with RV systolic pressure ≥40 mmHg and <40 mmHg, respectively), suggesting that elevated LV filling pressure contributed to pulmonary hypertension along with parenchymal lung disease and pulmonary vascular disease [33].

ECHOCOVID-19 is a prospective multicentre cohort study, including 214 consecutive COVID-19 patients, hospitalized in Denmark. COVID-19 patients (cases) were matched 1:1 with controls from the general population on the basis of age, sex and history of hypertension. In this study, no differences were found between cases and controls regarding LVEF, but LV global longitudinal strain (GLS) was significantly reduced in COVID-19 patients [35]. GLS, measured using two-dimensional speckle tracking echocardiography, is more sensitive than LVEF in the evaluation of LV systolic function and is often reduced also in patients with HFpEF [50]. A pattern of reduced basal LV longitudinal strain was observed in more than a half of hospitalized COVID-19 patients, undergoing speckle-tracking echocardiography in different studies [51, 52]. Patients with reduced basal longitudinal strain were more likely to have concomitant hypertension, obesity and diabetes [51]. Also RV systolic function, as assessed by RV longitudinal strain and tricuspid annular plane systolic excursion (TAPSE), was reduced in COVID-19 patients [35]. A more pronounced impairment in LV-GLS and RV longitudinal strain was reported in those with a severe form of COVID-19. Both LV-GLS and RV longitudinal strain were found to be independent predictors of mortality even after adjusting for multiple potential confounders [34, 35, 53].

In a systematic review of patients with COVID-19 who underwent cardiac magnetic resonance (CMR), signs of myocardial inflammation and injury including T1 and T2 abnormalities, oedema on T2/STIR and late gadolinium enhancement (LGE) were present in the majority of patients, despite normal LV systolic function [36•]. Importantly, CMR studies confirmed that fibrosis and oedema were predominantly located in the basal and mid LV segments [43]. The most plausible hypothesis is that basal regions are more susceptible to systemic stressors leading to myocardial damage [51].

The mechanisms behind acute myocardial damage are multiple. First, non-specific process, including respiratory failure, hypoxemia, systemic inflammatory response and coagulation disorders, may play a role in the genesis of myocardial damage [4•]. Second, SARS-CoV-2 binds angiotensin converting enzyme-2 (ACE-2) as host cellular receptor [54]. ACE-2 is expressed in different tissues, including fibroblasts, cardiomyocytes and endothelial cells [55]. Thus, the virus may enter cardiomyocytes causing direct damage [4•]. SARS-CoV-2 positivity in cardiac tissue has been described in endomyocardial biopsies of patients with suspected myocarditis or unexplained HF [37–39]. Of note, an analysis of 39 consecutive autopsies revealed viral genome in myocardial tissue of patients who did not manifest clinically evident CV issues [56]. It’s still not known if myocardial viral activity, even in the absence of clinical manifestations, might result in long-term consequences.

## After COVID-19: Persistent Myocardial Dysfunction

Signs and sequelae of myocardial injury may persist even after the acute phase of COVID-19 (Table 2). LGE is the gold-standard method to quantify myocardial fibrosis [57, 58]. LGE is higher in patients with LV diastolic dysfunction (e.g. reduced E wave deceleration time) and is associated with elevation of LV filling pressure (E/e’ ratio) [59].

Among recovered COVID-19 patients presenting cardiac symptoms, CMR revealed cardiac involvement in 58% of cases, with myocardial oedema, fibrosis, and impaired right ventricular function [43]. Similar findings were also described in patients without CV symptoms. In a cohort of 100 unselected patients recovered from COVID-19, without previously known cardiomyopathy, 78% had abnormal CMR findings indicative of myocardial inflammation and damage, including raised native T1 and T2 measures, myocardial LGE and pericardial enhancement. Findings were irrespective of underlying comorbidities, severity of the acute illness, time from COVID-19 diagnosis. Signs of myocardial inflammation were found in 60% of the patients, although LV systolic function was generally preserved [40]. In a prospective cohort of 26 otherwise healthy athletes, who had an asymptomatic course or a mild form of COVID-19, 4 (15%) had CMR findings suggestive of myocarditis and 8 additional athletes (30.8%) exhibited LGE in the absence of T2 elevation [42].

Echocardiographic findings confirmed that some of COVID-19 patients, even without underlying comorbidities and not requiring hospitalization due to a mild form of the disease, experienced myocardial injury after recovery. In a cohort of 54 consecutive student athletes, recovering from COVID-19, a total of 6 (11%) patients had reduced GLS. Symptomatic COVID-19 athletes had significantly lower septal e’ and average e’ velocities, as compared to asymptomatics [41]. The recognition of such structural and functional abnormalities, in some way typical of diastolic HF, raised concerns that COVID-19 survivors may develop HFpEF in a long-term period.

## COVID-19 and HFpEF: Pathophysiological Pathways

**Fig. 1:**
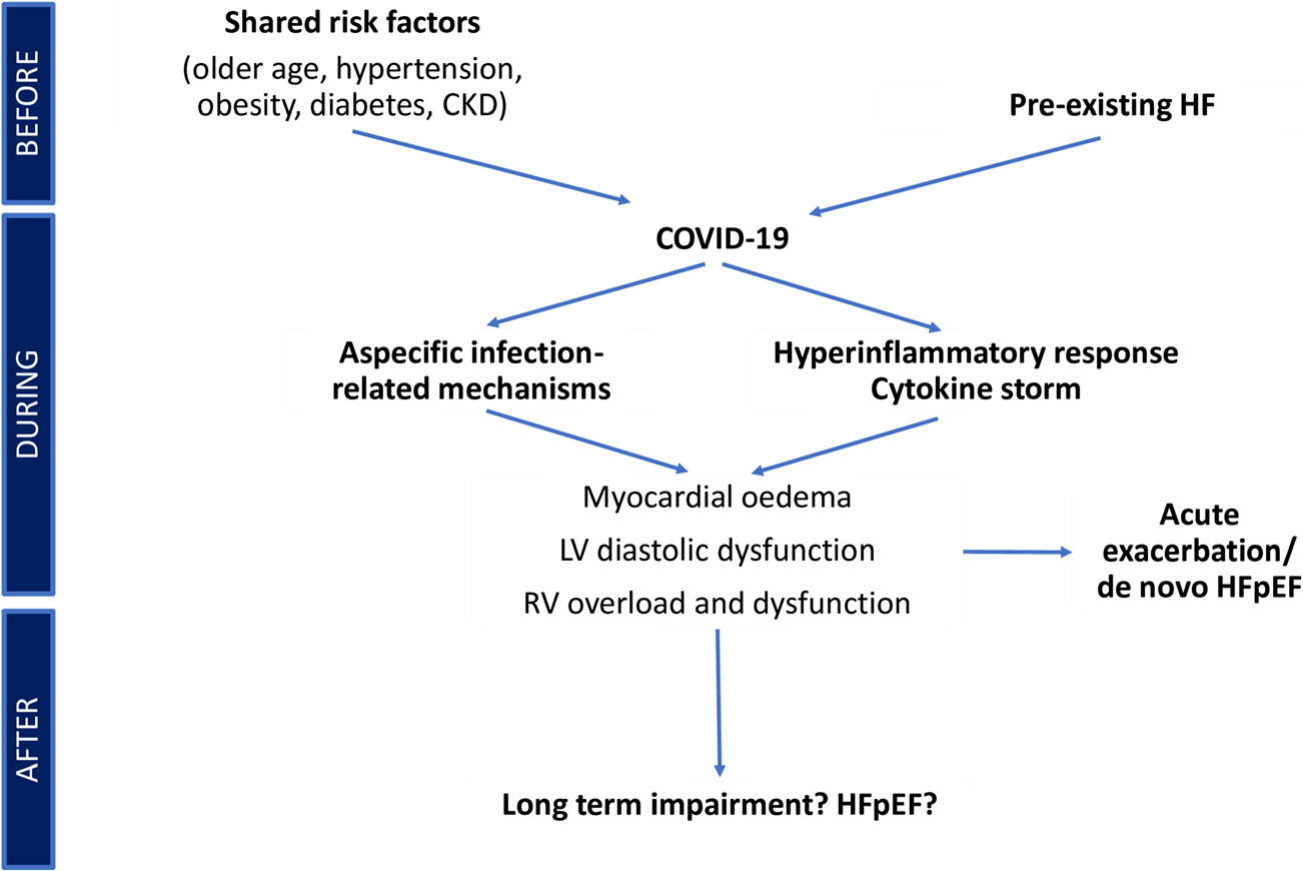
The short- and long-term relationship between COVID-19 and HFpEF. CKD, chronic kidney disease; COVID-19, Coronavirus Disease 2019; HF, heart failure; HFpEF, heart failure with preserved ejection fraction; LV, left ventricular; RV, right ventricular

COVID-19 may be associated with HFpEF through different pathways (Fig. 1). First, in patients with pre-existing HFpEF, COVID-19, as well as other respiratory infections, may be a simple precipitating factor leading to acute decompensation of HF. Second, elderly patients, with hypertension, diabetes, obesity or CKD — known risk factors for COVID-19 — have often LV hypertrophy and diastolic dysfunction even in the absence of overt HFpEF. COVID-19 may favour the clinical emergence of a subclinical disorder in these patients, partially explaining the high rates of de-novo HF onset. Third, COVID-19 might be a novel independent risk factor for the development of HFpEF, given the recent studies revealing the presence of abnormal myocardial structure and function in the acute phase of the disease and after recovery.

Inflammation, along with the shared cardiometabolic risk profile, has been suggested as the common link between COVID-19 and HFpEF [5]. Inflammation is a crucial mechanism in the pathogenesis of HFpEF [60]. Kalogeropoulos et al. demonstrated a significant association between increased levels of cytokine, including IL6, TNF-α, and the incidence of HFpEF but not HF with reduced ejection fraction (HFrEF) [61]. Obesity is characterized by a chronic low state of inflammation and cytokines produced in adipose tissue (TNF-α, IL1β, IL-6 and IL-15) are similar to those involved in COVID-19 [62, 63]. Similarly, diabetes, hypertension and CKD cause oxidative stress and consequent inflammation, representing a risk factor for CV disease [64–66]. Otherwise, in the acute phase of COVID-19, a storm of plasma cytokines and chemokines has been described, with higher markers of inflammation (e.g. C-reactive protein, ferritin, IL-2 IL-4, IL-6, IL-8, IL-10, TNF-α, and INF-γ) in patients with more severe presentations [8, 67, 68]. Inflammatory cytokine IL-6 was almost 10-fold increased in critically ill patients and was related to viral load (RNAemia) [69]. IL-6 was associated with platelet abnormalities and may induce activation of coagulation promoting a prothrombotic state [70]. COVID-19 patients with concomitant diabetes, obesity or CKD had also higher levels of inflammatory markers [71–73], suggesting a correlation between the burden of inflammation, comorbid conditions and disease severity.

Comorbidities leading to HFpEF induce a systemic proinflammatory state, which causes coronary microvascular endothelial dysfunction, reduction in nitric oxide bioavailability and subsequent LV stiffness and remodelling [74]. Similarly, COVID-19 inflammatory cardiomyopathy may result in LV remodelling and HF symptoms even with preserved LVEF [75].

## Conclusions

Cardiac involvement has been detected not only in the acute phase of COVID-19, but also in subacute or chronic phases. COVID-19 causes systolic dysfunction only in a minority of patients, while diastolic impairment, pulmonary hypertension, RV dysfunction, decrease in LV-GLS and RV longitudinal strain are common sequelae. COVID-19 inflammatory cardiomyopathy may represent a possible long term consequence of the disease, raising concerns about an increase in HF prevalence, namely HFpEF.
